# Simultaneous non-contact mapping fused with CMR derived grey zone to explore the relationship with ventricular tachycardia substrate in ischaemic cardiomyopathy

**DOI:** 10.1186/1532-429X-15-S1-P64

**Published:** 2013-01-30

**Authors:** Zhong Chen, Jatin Relan, Walther H Schulze, Rashed Karim, Manav Sohal, Anoop Shetty, YingLiang Ma, Nicholas Ayache, Maxime Sermesant, Herve Delingette, Julian Bostock, Reza Razavi, Kawal Rhode, Aldo Rinaldi

**Affiliations:** 1 Kings College London, London, UK; 2 INRIA Sophia Antipolis, Sophia Antipolis, France; 3 Karlsruhe Institute of Technology, Karlsruhe, Germany

## Background

Cardiac magnetic resonance (CMR) imaging enables characterization of myocardial scar and the ‘grey zone', an admixture of scar and healthy myocardium, which is an independent predictor of ventricular arrhythmia. We explored the relationship between the grey zone and ventricular tachycardia circuits (VT) in ischaemic cardiomyopathy.

## Methods

**Figure 1:**
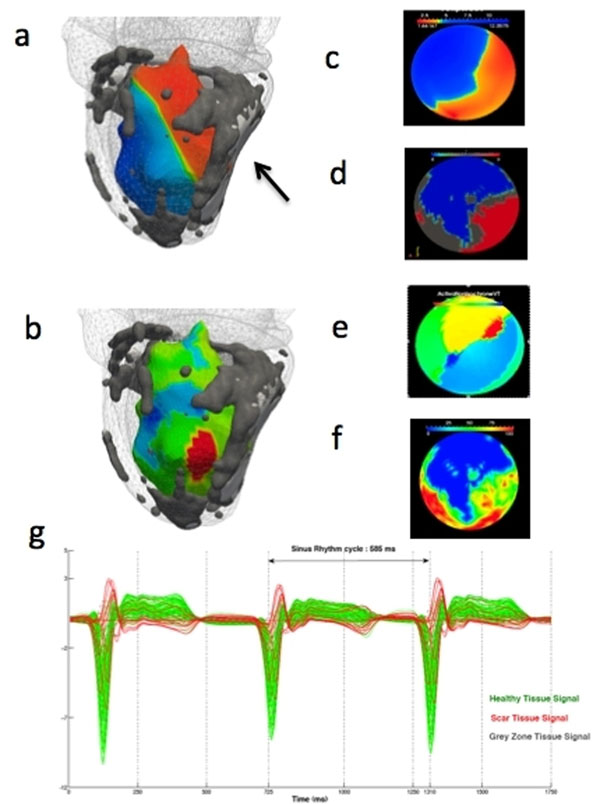
Correlation of EnSite electro-anatomical mapping with tissue heterogeneity assessment with CMR. a: MRI shell with scar core (white, shown by arrow) and grey zone (grey) superimposed on the EnSite shell illustrating the voltage potential map (blue = maximum voltage, red ≤ 30% of maximum voltage). b: MRI shell with scar distribution superimposed on the EnSite shell illustrating the isochrone map during VT (red = exit point). c: Bull's plot of EnSite substrate map during sinus rhythm (blue = maximum voltage, red ≤ 30% of maximum voltage). d: Bull's eye plot of endocardial scar distribution scar core (white), grey zone (grey) and healthy (black) from CMR. e: Bull's eye plot of isochrone map illustrating the locations of the earliest activation (red, exit point) and latest activation (blue) during VT. f: Bull's eye plot of transmural myocardial signal intensity variation on CE-CMR reflecting heterogeneity in myocardial tissue (blue = most homogenous transmural signals, likely to represent healthy myocardium; red = most heterogeneous transmural signals, likely to represent fibrotic tissue). g. Unipolar signal recording from MEA during 3 sinus rhythm beat (x-axis = time; y-axis =voltage).

Two patients with previous myocardial infarct underwent high-resolution late gadolinium enhanced CMR scar imaging (1.2x1.2x2.6mm) and a VT-stimulation study. The LV scar core was segmented using full-width-half-maximum method; and the grey zone was segmented with a cut-off signal intensity below that of the scar core and above 2 standard-deviation of the remote healthy myocardium. A multi-electrode array (MEA) was positioned in the LV cavity for simultaneous electroanatomical mapping during the study. The MEA shell was registered with the CMR-derived LV shell using anatomical landmark registration (Figure 1a,b) for comparison.

## Results

Sustained monomorphic VT (SMVT) was induced in both patients. Scar core and grey zone regions correlated well with the low voltage area (≤ 30% maximum voltage) seen on the MEA (Figure 1a,c,d). The exit point during SMVT from the isochrone map was located close to a channel of healthy myocardium within the grey zone (Figure 1b,d,e,f). The unipolar signals have distinct peak negative voltage in regions of scar core, grey zone and healthy myocardium (Figure 1g).

## Conclusions

We have demonstrated that the critical substrate of re-entrant SMVT lies within the grey zone in the two ischemic cardiomyopathy study cases. CMR characterization of scar heterogeneity may provide useful information predicting the location of the critical substrate in re-entrant VT observed in patients with a previous history of myocardial infarction.

## Funding

N/A

